# Supplementary material for: Developmental Neurotoxicity of Environmentally Relevant Pharmaceuticals and Mixtures Thereof in a Zebrafish Embryo Behavioural Test
Source: Int J Environ Res Public Health. 2021 Jun 22;18(13):6717. doi: 10.3390/ijerph18136717 (PMC8297305; doi:10.3390/ijerph18136717)
Supplement: Supplementary file 1 [file ijerph-18-06717-s001.zip › ijerph-1238960-SI.pdf]

Supplementary data

to: Developmental neurotoxicity of mixtures of environmentally relevant pharmaceuticals and mixtures thereof in a zebrafish embryo behavioral test

Alessandro Atzei, Ingrid Jense, Edwin P. Zwart, Jessica Legradi, Bastiaan J. Venhuis, Leo T. M. van der Ven, Harm J. Heusinkveld, Ellen V. S. Hessel.

Contents

ZFET dose-range 2
GMS DR curves of FLX and norFLX 3
Carbamazepine 4
Fluoxetine 5
Venlafaxine 6
Carbamazepine BMD confidence intervals 7
Fluoxetine BMD confidence intervals 8
Venlafaxine BMD confidence intervals 9
Overview single-compound DR analyses 10
Phenytoin 11
Exposure windows analysis 12

ZFET dose-range

Table S1. Test compounds and exposure dose-ranges in ZFET

Compound	Code	CAS	Dose-range (µM)
Carbamazepine	CBZ	298-46-4	0, 5, 25, 50, 250
Carbamazepine 10,11-epoxide	CBZ10,11E	36507-30-9	0, 0.003, 0.03, 0.35, 3.5, 35
Fluoxetine	FLX	54910-89-3	0, 0.0089, 0.089, 0.89, 8.9, 89.9
Norfluoxetine	norFLX	57226-68-3	0, 0.006, 0.06, 0.6, 6, 60
Phenytoin	PHT	57-41-0	0,3,10,30,100,300
Venlafaxine	VNX	93413-69-5	0, 0.03, 0.3, 3.18, 31.86, 318.61
Desvenlafaxine	desVNX	300827-87-6	0, 0.03, 0.33, 3.33, 33.35, 333.51

GMS DR curves of FLX and norFLX

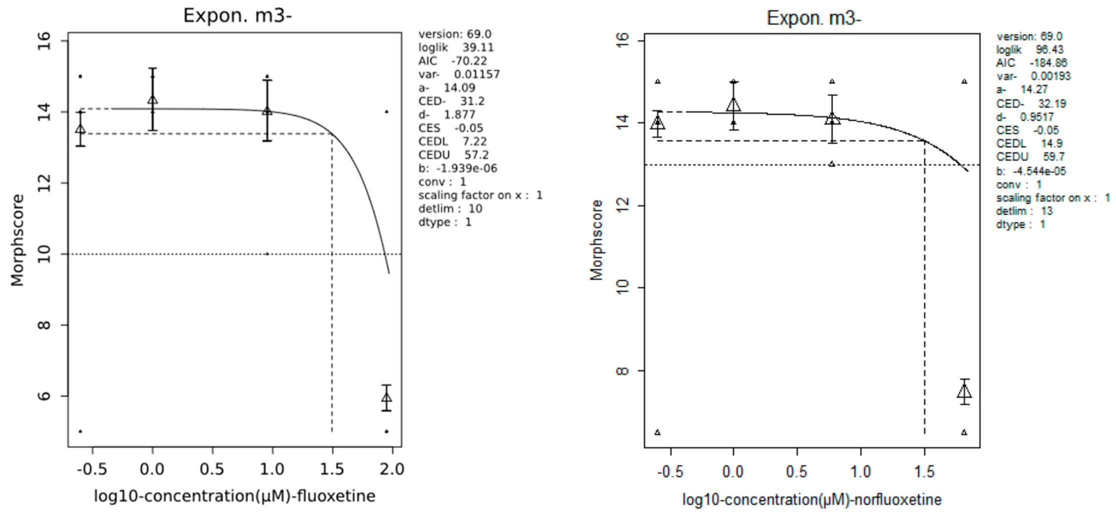

Fig S1. Dose-response curves of general morphology score performed at 72 hpf for fluoxetine and norfluoxetine. X- axis shows compounds dose-range on log scale.

Carbamazepine

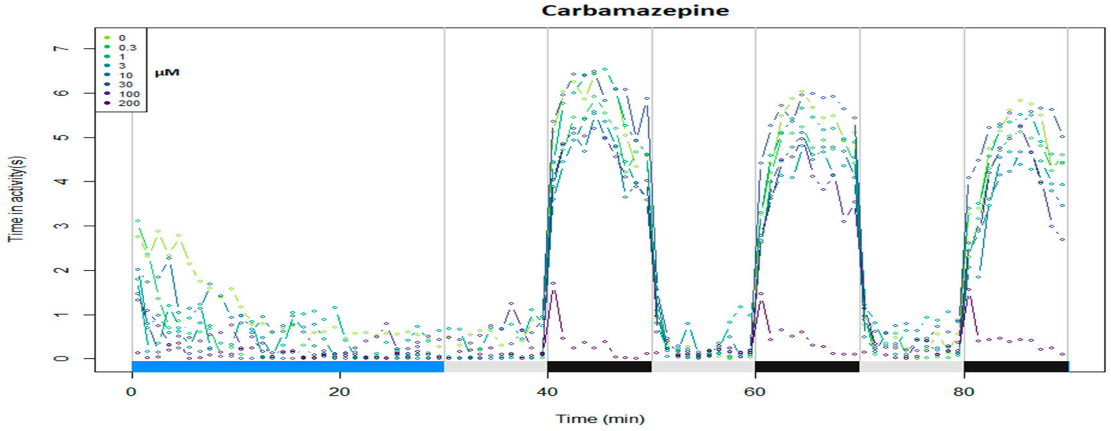

Fig. S2. Plot overview showing a complete light-dark transition test applied for the CBZ experiment, after 0-120hpf exposure. This experiment was performed at the RIVM.

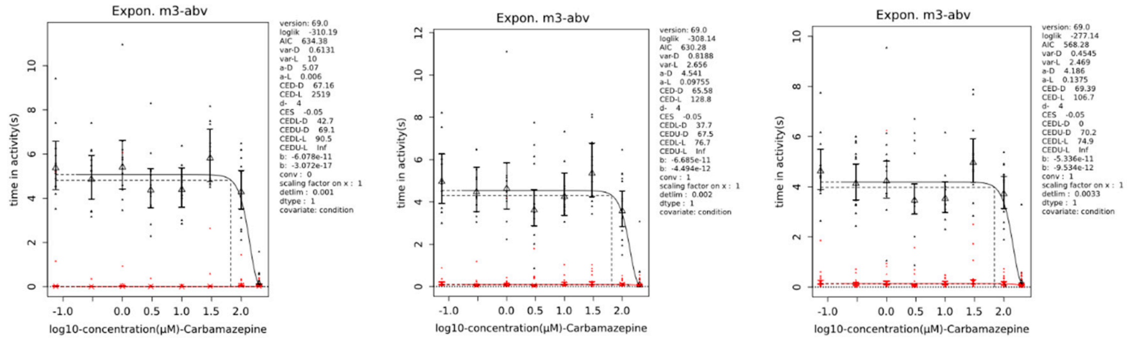

Fig. S3 Dose-response curves of an individual CBZ experiment. The data set belong to the second and third light-dark block, respectively. This experiment (plot overview and first block reported in the publication) was performed at the RIVM.

Table S2. Carbamazepine CEDs at 5% effect level of each dark period of the L-D transition test.

Block 1	Block 2	Block3
CED_{05}(CI) (µM)	CED_{05}(CI) (µM)	CED_{05}(CI) (µM)
67.16 (42.7-69.1)	65.58 (37.7-67.)	69.39 (47.1-71.3)

Fluoxetine

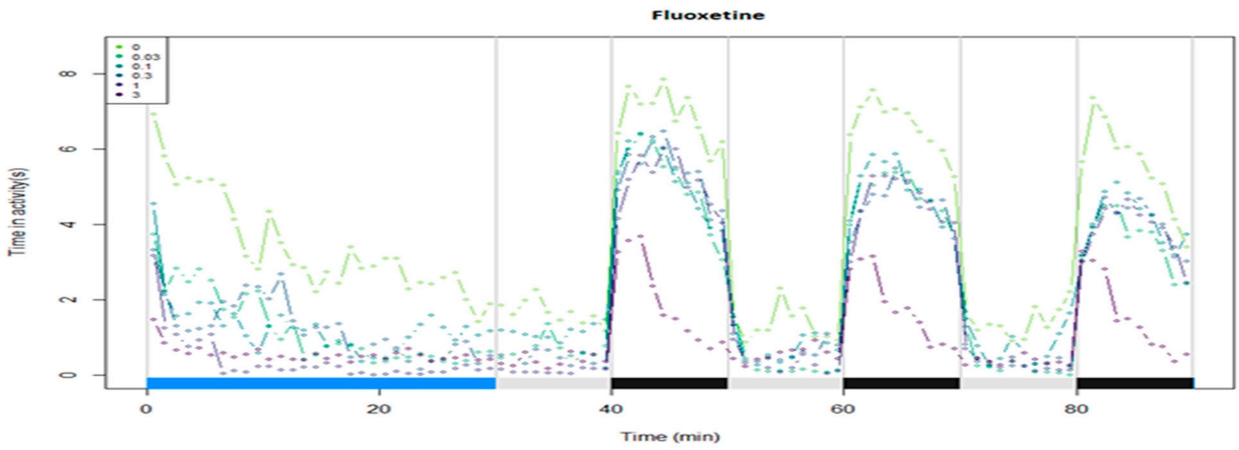

Fig. S4. Plot overview showing a complete light-dark transition test applied for the FLX experiment, after 0-120hpf exposure. This experiment was performed at the RIVM.

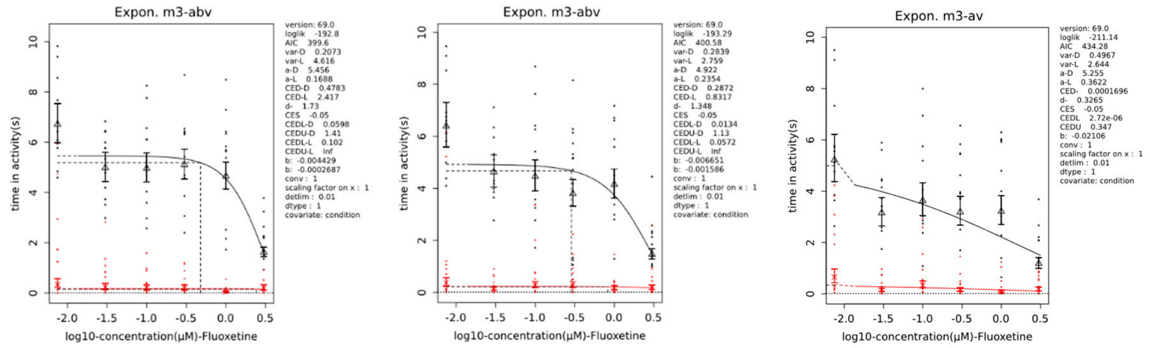

Fig. S5. Dose-response curves of an individual FLX experiment. The data set belong to the first, second and third light-dark block, respectively.

Table S3. Fluoxetine CEDs at 5% effect level of each dark period of the L-D transition test.

Block 1	Block 2	Block3
CED_{05}(CI) (µM)	CED_{05}(CI) (µM)	CED_{05}(CI) (µM)
0.48 (0.06-1.41)	0.29 (0.01-1.13)	0.0002 (0.00003-0.35)[a]

[a] Block 3 response deviates from block 1-2 (similar with VNX, but different from CBZ), leading to a much lower CED but with a very wide CI. Block 3 CED is therefore less reliable.

Venlafaxine

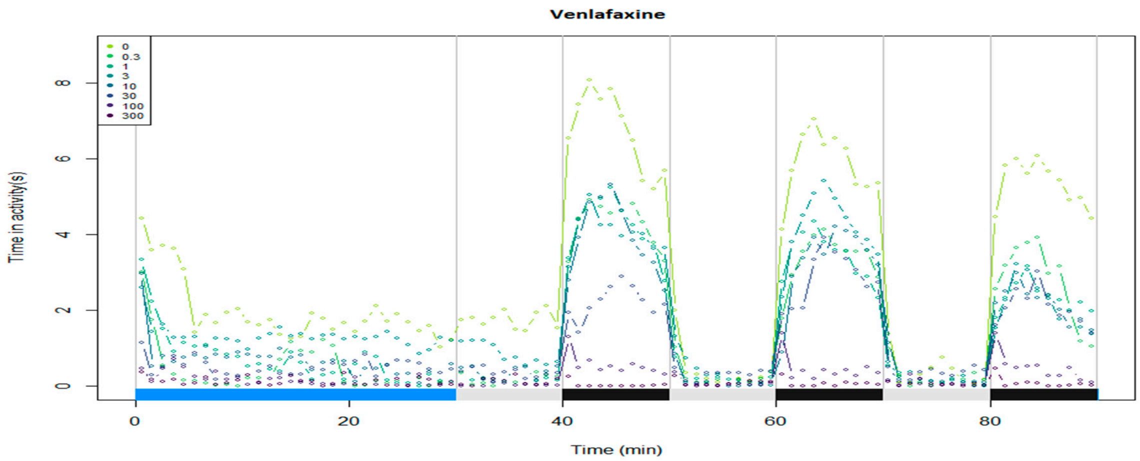

Fig. S6. Plot overview showing a complete light-dark transition test applied for the VNX experiment, after 0-120hpf exposure. This experiment was performed at the RIVM.

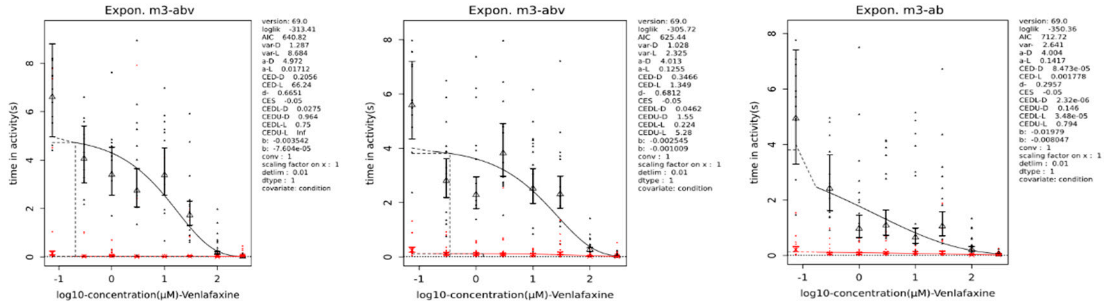

Fig. S7. Dose-response curves of an individual VNX experiment. The data set belong to the first, second and third light-dark block, respectively.

Table S4. Venlafaxine CEDs at 5% effect level of each dark period of the L-D transition test.

Block 1	Block 2	Block3
CED_{05}(CI) (µM)	CED_{05}(CI) (µM)	CED_{05}(CI) (µM)
0.21 (0.028-0.96)	0.35 (0.05-1.35)	0.000084 (0.0000023-0.15)[a]

[a] Block 3 response deviates from block 1-2 (similar with FLX, but different from CBZ), leading to a much lower CED but with a very wide CI. Block 3 CED is therefore less reliable.

Carbamazepine BMD confidence intervals

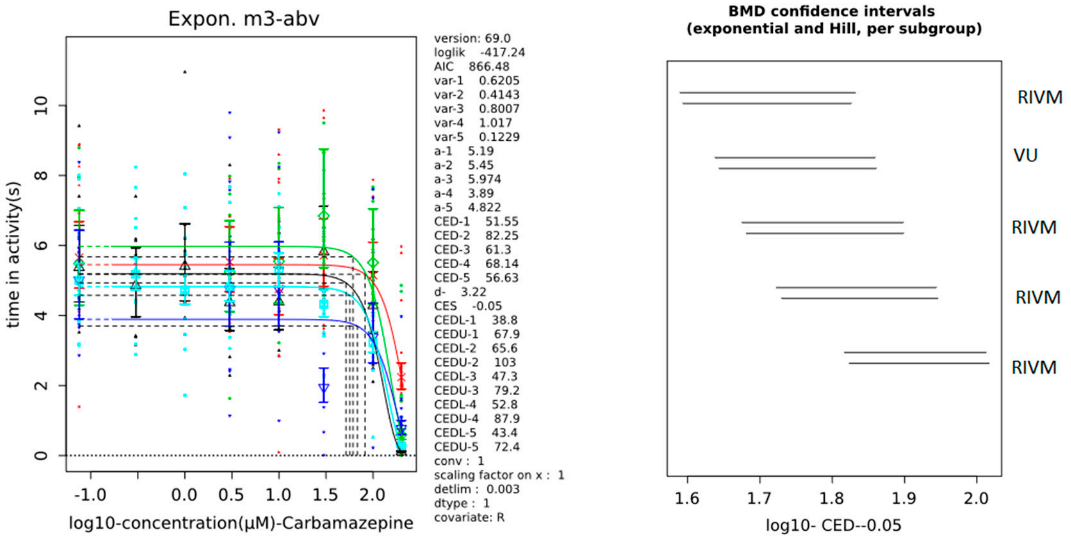

Fig. S8. Distribution of BMDs over the five repeated experiments of CBZ. Each pair of lines represents the confidence interval (CI) of an independent experiment, related to the exponential (upper) and Hill (lower) model. X-axis, log10 of concentration in µM. The five experiments produce one overlapping cluster, within a combined range of 51.55 to 82.25 µM

Fluoxetine BMD confidence intervals

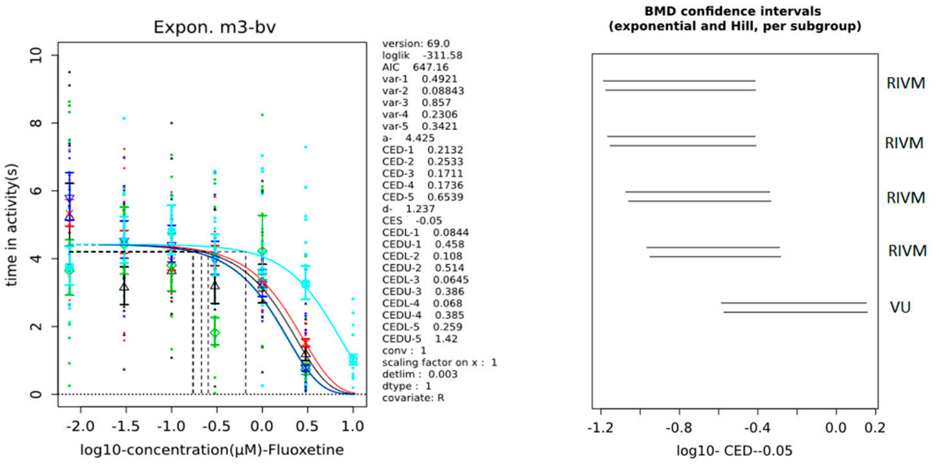

Fig. S9. Distribution of BMDs over the five repeated experiments of **FLX.** Each pair of lines represents the confidence interval (CI) of an independent experiment, related to the exponential (upper) and Hill (lower) model. X-axis, log10 of concentration in µM. The five experiments produce one cluster of overlapping CED-CIs, ranging from 0.17 to 0.65 µM.

Venlafaxine BMD confidence intervals

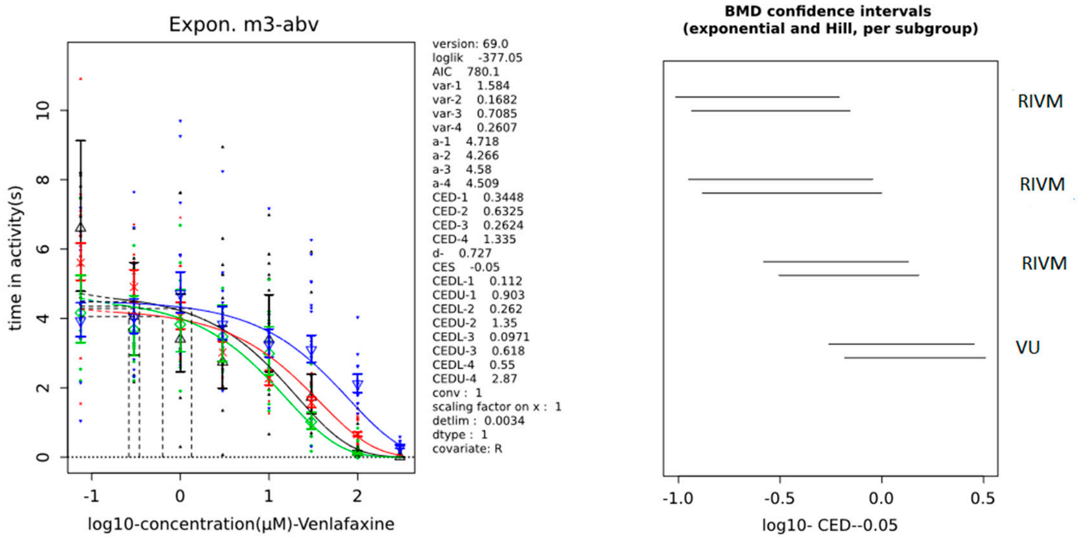

Fig. S10. Distribution of BMDs over the four repeated experiments of VNX. Each pair of lines represents the confidence interval (CI) of an independent experiment, related to the exponential (upper) and Hill (lower) model. X-axis, log10 of concentration in µM. The four experiments produce one cluster of overlapping CED-CIs, ranging from 0.26 to 1.34 µM.

Overview single-compound DR analyses

Table S5. Overview of replicate results in dark blocks with the three key reference compounds

Compound	CED$_{05}$ (CI)[a] µM per Block			location
	1	2	3	
**CBZ**	45.87 (33-63.3)	43.96 (28.4-66.9)	39.55 (24.3-61.7)	VU
	67.16 (42.7-69.1)	65.58 (37.8-67.8)	69.39 (47.1-71.3)	RIVM
	78.4 (43.7-80.6)	nd	nd	RIVM
	82.25 (52.5-103)	nd	nd	RIVM
	75.24 (45.2-85.8)	nd	nd	RIVM
**FLX**	0.79 (0.1-4.22)	1.27 (0.24-4.61)	1.84 (0.29-4.53)	VU
	0.48 (0.06-1.41)	0.29 (0.01-1.29)	0.0002 (0.00003-0.35)	RIVM
	0.17 (0.06-0.2)	nd	nd	RIVM
	0.47 (0.2-0.49)	nd	nd	RIVM
	0.18 (0.04-0.6)	nd	nd	RIVM
**VNX**	10.43 (3.61-25.5)	13.3 (4.47-34.2)	22.41 (6.14- 74.8)	VU
	0.21 (0.028-0.96)	0.35 (0.05-1.35)	0.000084 (0.0000023-0.15)	RIVM
	0.26 (0.01-2.01)	nd	nd	RIVM
	0.69 (0.12-2.74)	nd	nd	RIVM

[a] CEDs/CIs are given for exponential model analysis only. nd, not measured. Reported values are from separately analysed experiments and my thus deviate from results through analysis of combined analysis using “experiment” as covariate (Figs. S8-10), where the per-experiment fits are tweaked towards an overall model.

Phenytoin

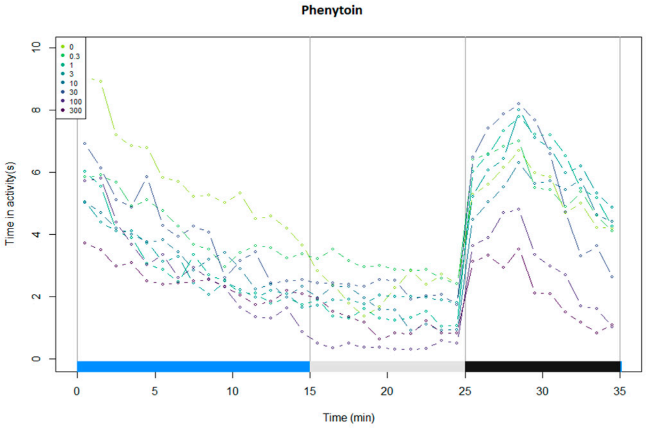

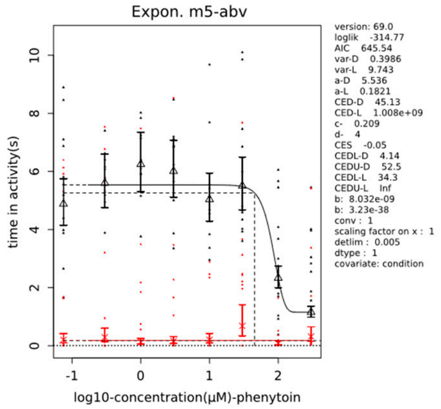

Fig. S11. Plot overview showing a short light-dark transition test applied for a PHT experiment (R1), after 0-120hpf exposure. This experiment was performed at the RIVM.

Fig. S12. A dose-response curve of an individual PHT experiment (R1). The data set belong to the first (and only) light-dark block.

Table S6. Phenytoin CEDs at 5% final effect (dark period)

R 1
CED_{05}(CI) (µM)
45.13 (4.14-52.5)

Exposure windows analysis

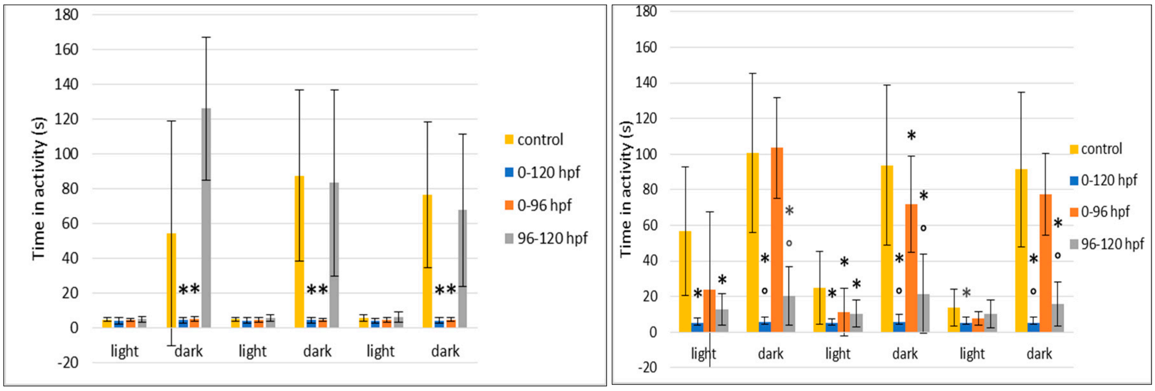

Figure S13. Activity measurement during six intermitted 10 min periods of light and dark (three periods each) at 96 hpf (A) and 120 hpf (B) after different times of exposure to 200 µM CBZ. The activity (Y-axis) was measured (s) as duration of movement each 10 min. The X-axis represents the light and dark periods. The asterisk indicates a significant difference compared to the control group and the circle indicates significance compared to the activity of the 0-96 hpf group, P<0.05. N=12

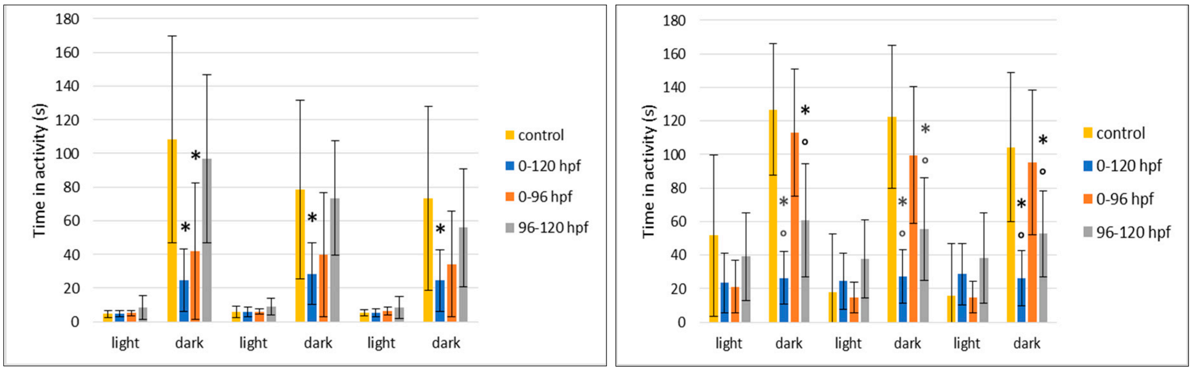

Figure S14. Activity measurement during six intermitted 10 min periods of light and dark (three periods each) at 96 hpf (A) and 120 hpf (B) after different times of exposure to 10 µM FLX. The activity (Y-axis) was measured (s) as duration of movement each 10 min. On the X-axis represents the light and dark periods. The asterisk indicates a significant difference compared to the control group and the circle indicates significance compared to the activity of the 0-96 hpf group, P<0.05. N

Exposure windows analysis

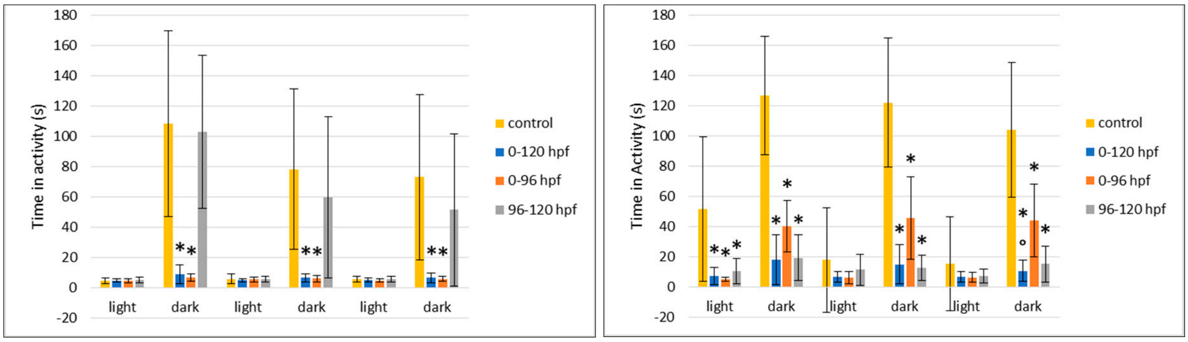

Figure S15. Activity measurement during six intermitted 10 min periods of light and dark (three periods each) at 96 hpf (A) and 120 hpf (B) after different times of exposure to 300 µM VNX. The activity (Y-axis) was measured (s) as duration of movement each 10 min. The X-axis represents the light and dark periods. The asterisk indicates a significant difference compared to the control group and the circle indicates significance compared to the activity of the 0-96 hpf group, P<0.05. N=12
